# Sonobiopsy for enrichment of circulating microRNAs in glioma patients

**DOI:** 10.1093/noajnl/vdag136

**Published:** 2026-05-22

**Authors:** Jinyun Yuan, Yuanxiang Li, Chih-Yen Chien, Lu Xu, Andrew H Stark, Siaka Fadera, Zhaoning Gu, Yu Xuan Chin, Kevin Xu, Arash Nazeri, Umeshkumar Athiraman, Xiaowei Wang, Hong Chen, Eric C Leuthardt

**Affiliations:** Department of Neurosurgery, Washington University School of Medicine, St. Louis, MO, USA; Department of Pharmacology and Regenerative Medicine, University of Illinois at Chicago, Chicago, IL, USA; Department of Neurosurgery, Washington University School of Medicine, St. Louis, MO, USA; Department of Biomedical Engineering, Washington University in St. Louis, St. Louis, MO, USA; Department of Biomedical Engineering, Washington University in St. Louis, St. Louis, MO, USA; Department of Biomedical Engineering, Washington University in St. Louis, St. Louis, MO, USA; Department of Neurosurgery, Washington University School of Medicine, St. Louis, MO, USA; Department of Neurosurgery, Washington University School of Medicine, St. Louis, MO, USA; Department of Neurosurgery, Washington University School of Medicine, St. Louis, MO, USA; Department of Biomedical Engineering, Washington University in St. Louis, St. Louis, MO, USA; Mallinckrodt Institute of Radiology, Washington University School of Medicine, St. Louis, MO, USA; Department of Anesthesiology, Washington University School of Medicine, St. Louis, MO, USA; Department of Pharmacology and Regenerative Medicine, University of Illinois at Chicago, Chicago, IL, USA; Department of Neurosurgery, Washington University School of Medicine, St. Louis, MO, USA; Department of Biomedical Engineering, Washington University in St. Louis, St. Louis, MO, USA; Division of Neurotechnology, Department of Neurosurgery, Washington University School of Medicine, St. Louis, MO, USA; Department of Neurosurgery, Washington University School of Medicine, St. Louis, MO, USA; Department of Biomedical Engineering, Washington University in St. Louis, St. Louis, MO, USA; Division of Neurotechnology, Department of Neurosurgery, Washington University School of Medicine, St. Louis, MO, USA; Department of Neuroscience, Washington University School of Medicine, St. Louis, MO, USA; Center for Innovation in Neuroscience and Technology, Washington University School of Medicine, St. Louis, MO, USA; Department of Mechanical Engineering and Materials Science, Washington University in St. Louis, St. Louis, MO, USA

**Keywords:** blood-brain barrier, focused ultrasound, glioma, liquid biopsy, microRNA

## Abstract

**Background:**

Sonobiopsy is a promising new technique that employs focused ultrasound (FUS) to noninvasively enrich brain tumor-derived biomarkers from spatially targeted brain location into the bloodstream. Building upon our recent first-in-human sonobiopsy trial demonstrating enrichment of circulating tumor DNA (ctDNA), here we investigated whether sonobiopsy can enhance the release and detection of tumor-derived microRNAs (miRNAs).

**Methods:**

Eleven patients with glioma underwent FUS sonication immediately prior to surgical resection. Peripheral blood samples were collected 5 min before and 5, 10, and 30 min after sonication. Plasma and brain tissue miRNA levels were quantified through small RNA sequencing and compared among different time points.

**Results:**

Plasma miRNAs that were below the detection threshold [reads per million (RPM) <10] showed significant enrichment at all post-FUS time points across all patients, with a maximal increase of 8.7-fold at 30 min. In contrast, miRNAs above the detection threshold (RPM ≥10) exhibited no significant change following FUS. Among the miRNAs that were initially below the detection threshold but increased to detectable levels following sonobiopsy, miR-29c-5p, miR-125b-1-3p, miR-129-5p, miR-132-3p, miR-143-5p, miR-149-5p, miR-195-5p, miR-218-5p, miR-329-3p, and miR-1271-5p were associated with cancer- and glioma-related biological pathways.

**Conclusions:**

These findings extend our prior work and support sonobiopsy as a spatially targeted, noninvasive multimodal liquid biopsy platform. By increasing the detectability of otherwise difficult-to-detect tumor-derived signals, sonobiopsy has the potential to advance the development of sensitive molecular diagnostics for glioma without surgery.

Key Points

- Sonobiopsy selectively enriches plasma miRNAs that are below the detection threshold
- Sonobiopsy does not significantly increase plasma miRNAs that are above the detection threshold
- Sonobiopsy-enriched miRNAs are associated with cancer- and glioma-related pathways

Importance of the Study

Sensitive detection of circulating biomarkers remains a major barrier to liquid biopsy for brain tumor diagnosis and monitoring, largely due to the restrictive blood-brain barrier (BBB). Although focused ultrasound (FUS) can transiently increase BBB permeability, its potential to improve liquid biopsy sensitivity has only been explored recently. Our group previously conducted the first-in-human sonobiopsy trial, demonstrating that FUS can enrich circulating tumor DNA (ctDNA) in glioma patients. However, whether sonobiopsy can similarly enhance the detection of circulating microRNAs (miRNAs), a biologically relevant brain tumor biomarker, has not been investigated.

This study provides the first clinical evidence that sonobiopsy selectively enriches circulating miRNAs in glioma patients. We show that plasma miRNAs below the threshold become consistently and significantly elevated after FUS. By integrating small RNA sequencing of tumor and plasma, we identify a reproducibly enriched miRNA signature with established relevance to glioma biology. These findings expand sonobiopsy beyond ctDNA, establishing it as a multimodal liquid biopsy platform capable of overcoming the low circulating abundance of brain tumor biomarkers.

Together with prior studies, our results indicate that sonobiopsy can substantially improve the sensitivity of blood-based diagnostics for brain tumors by enabling spatially targeted, noninvasive release of tumor-derived nucleic acids. This approach has important implications for diagnosis, treatment monitoring, recurrence detection, and precision neuro-oncology. Future studies should evaluate multimodal biomarker enrichment and assess the clinical utility of sonobiopsy-enriched analytes.

Liquid biopsy has transformed the diagnosis and management of many peripheral cancers by enabling minimally invasive molecular profiling through analysis of circulating tumor-derived biomarkers. However, its application to brain tumors has been limited. In patients with glioma, circulating biomarkers, including nucleic acids, proteins, and extracellular vesicles, are often detected at extremely low levels and only in a subset of individuals.^1^^,^^2^ This scarcity arises largely from the blood-brain barrier (BBB), which restricts the transport of tumor-derived materials from the central nervous system into peripheral circulation.^3^^,^^4^ As a result, even the most sensitive detection platforms, such as droplet digital PCR and next-generation sequencing, frequently struggle to capture brain tumor-derived signals with sufficient reliability for clinical use.^5^ A further constraint of conventional liquid biopsy is the inability to determine the anatomical origin of detected biomarkers.

Focused ultrasound (FUS) combined with intravenously administered microbubbles enables safe, localized, and reversible opening of the BBB.^6^ Initially developed to enhance drug delivery, FUS-medicated BBB opening (FUS-BBBO) has been clinically validated as safe and feasible in patients with brain tumors.^7^ Beyond permitting drug entry, our group and others have shown that FUS-BBBO can also promote the outward movement of brain-derived molecules into the bloodstream,^8-10^ a technique we have termed sonobiopsy.^11^ Sonobiopsy uses ultrasound to enrich circulating biomarkers from ultrasound-targeted disease regions, enabling sensitive and spatially targeted molecular diagnosis via liquid biopsy. Early clinical studies in glioma patients have demonstrated that sonobiopsy increases the detectability of multiple classes of tumor-derived biomarkers, including circulating cell-free DNA (cfDNA), circulating tumor DNA (ctDNA), extracellular vesicles, and proteins.^8-10^^,^^12^

MicroRNAs (miRNAs) are an especially compelling class of circulating biomarkers. These short noncoding RNAs (typically 19–22 nucleotides long) regulate post-transcriptional gene expression and contribute to virtually all hallmarks of cancer, including proliferation, invasion, metabolic reprogramming, immune evasion, and angiogenesis.^13-15^ In gliomas, dysregulated miRNA expression patterns have been consistently observed, with both tumor-suppressive and oncogenic miRNAs linked to tumor grade, progression, therapeutic response, and patient prognosis.^16^^,^^17^ Compared with DNA, miRNAs exhibit exceptional stability in blood because they are naturally protected from RNase degradation.^18^ However, despite a strong biological rationale, circulating miRNAs in glioma patients have remained difficult to detect due to their low detection in peripheral blood.^19^ Consequently, miRNAs are widely recognized as one of the most promising yet inaccessible biomarkers for brain tumors.^20^^,^^21^

This unmet need highlights the importance of strategies that increase the availability of tumor-derived miRNAs in circulation, rather than relying solely on improvements in analytical sensitivity. Sonobiopsy offers this approach by enabling spatially targeted, temporally controlled release of tumor-derived miRNAs into the bloodstream, thereby converting previously undetectable signals into measurable biomarkers. Prior work in a rat model of prostate cancer suggested that FUS can increase circulating miRNA levels; however, that study employed high-pressure pulsed ultrasound to induce cavitation, thereby permeabilizing or liquifying tumor tissue,^22^ which is not a clinically translatable parameter for safe BBB opening. In contrast, low-intensity FUS combined with microbubbles can induce safe, transient and reversible BBB opening, thereby providing a noninvasive and targeted method for enriching tumor-derived nucleic acids in patients.

Here, we present a prospective clinical study evaluating whether sonobiopsy can enhance the release and detection of circulating tumor-derived miRNAs in patients with glioma. We show that sonobiopsy selectively enriches tumor-derived miRNAs that were below threshold in plasma—precisely those most restricted by the BBB and least detectable under pre-FUS conditions. These findings demonstrate that miRNAs, long viewed as promising but difficult-to-detect biomarkers in neuro-oncology, can be sensitively and noninvasively enriched using sonobiopsy. This work supports the further development of sonobiopsy as a spatially targeted strategy to improve molecular diagnostics and enable multi-analyte liquid biopsy platforms for glioma.

## Methods

### Study Design

This prospective, single-arm, single-center, first-in-human study was designed to evaluate the feasibility and safety of sonobiopsy in patients with brain tumors. Patients with brain lesions with imaging characteristics consistent with glioma were screened for the clinical trial. Details of the inclusion and exclusion criteria are provided in Table S1.

A total of 11 patients with glioma were enrolled (Table 1), including 7 males and 4 females with a median age of 63 years (range: 36-73). The cohort included patients with glioblastoma (*n* = 7), diffuse high-grade glioma (*n* = 2), astrocytoma (*n* = 1), and oligodendroglioma (*n* = 1). IDH1 R132H mutation status was predominantly wild type (*n* = 9), with two patients harboring IDH1 mutations. Overall, the demographic and molecular characteristics reflect a representative adult glioma population.

**Table 1.** Patient demographics and key ultrasound parameters

Patient	Sex	Age	Diagnosis	IDH1	Simulated in situ peak negative pressure (MPa)	Estimated mechanical index (MI)
1	M	47	Astrocytoma	IDH1R132H	0.46	0.57
2	F	51	Oligodendroglioma	IDH1R132H	0.86	1.07
3	M	69	Glioblastoma	Wild type	0.66	0.82
4	M	66	Glioblastoma	Wild type	0.53	0.66
5	M	36	Diffuse high-grade glioma	Wild type	0.40	0.50
6	F	63	Glioblastoma	Wild type	0.34	0.42
7	M	69	Glioblastoma	Wild type	0.30	0.37
8	F	73	Glioblastoma	Wild type	0.32	0.40
9	M	53	Glioblastoma	Wild type	0.28	0.40
10	F	63	Diffuse high-grade glioma	Wild type	0.15	0.18
11	M	69	Glioblastoma	Wild type	0.30	0.37

### Sonobiopsy Device

Sonobiopsy was performed using a 650-kHz FUS transducer composed of 15 concentric ring elements (Imasonics, Voray-sur-l’Ognon, France). The FUS transducer was driven by an electronic transmitting system (Image Guided Therapy, Pessac, France). A water bladder was attached to the FUS transducer with an inlet and outlet for degassing the water. The transducer has a focal length of 65 mm and generates a focal spot with axial and lateral full-width at half-maximum dimensions of approximately 20 mm and 3 mm, respectively. The FUS transducer was mechanically aligned with a Medtronic Stealth S8 neuronavigation probe using a custom adapter, enabling image-guided targeting. An 80-mm offset was applied in the neuronavigation software to align the virtual probe tip with the FUS focus.

### Sonobiopsy Procedure

**Figure 1.**
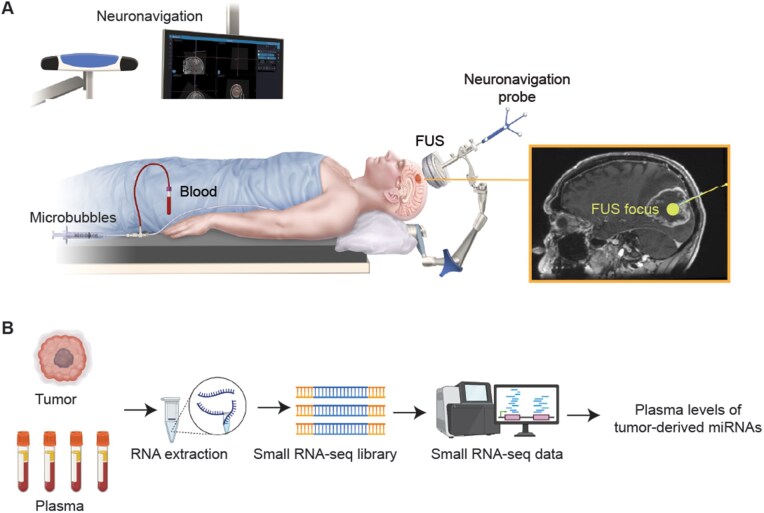
Schematic of sonobiopsy setup and plasma miRNA analysis pipeline. (A) Neuronavigation-guided FUS is applied to the tumor region immediately before surgical resection. After placement of the neuronavigation probe and confirmation of the FUS target, microbubbles were administered intravenously, and the FUS sonication was subsequently performed. Peripheral blood samples were collected before and at multiple time points after sonication for downstream analysis. (B) Plasma and tissue samples undergo RNA extraction, followed by small RNA library preparation and high-throughput sequencing. Sequencing data are processed through a bioinformatics pipeline including alignment, counting, and normalization to quantify plasma levels of tumor-derived miRNAs. Panel (A) was adapted from Yuan, *npj Precision Oncology*, 2023, under the Creative Commons Attribution 4.0 International License. Panel (B) was created in BioRender. Chen, H. (2026) https://BioRender.com/p8y2btm.

The clinical workflow and FUS device used for sonobiopsy have been described previously (Figure 1).^12^ In brief, CT and MRI scans were acquired to plan the sonication trajectory using the Medtronic Stealth S8 system (Medtronic, Minneapolis, MN, USA). Trajectories were selected to optimize acoustic incidence angle, maintain an appropriate focal depth, and avoid critical structures such as the eye and ear canal. After induction of anesthesia, patients were positioned in a Mayfield skull clamp (Integra LifeSciences, Princeton, NJ, USA) and registered to preoperative imaging. A small patch of hair above the tumor region was shaved, and the exposed skin was thoroughly cleaned with alcohol pads. Degassed ultrasound gel was applied liberally to the exposed skin area. The FUS transducer was then placed on the patient’s head under neuronavigation guidance, allowing the acoustic focus to be projected along the preplanned trajectory. Sonication parameters were: 650 kHz center frequency, 1 Hz pulse repetition frequency, 10 ms pulse duration, and 3 min total duration. Microbubbles (Definity, Lantheus Medical Imaging, North Billerica, MA) were injected intravenously at a dose of 10 µL/kg body weight to facilitate BBB opening. The FUS acoustic pressure values at the focus were simulated based on the probe’s final trajectory using the k-Wave toolbox as reported in our previous publication (Table 1).

Peripheral blood samples (20 mL) were drawn 5 min before FUS and at 5, 10, and 30 min after FUS. Samples were stored on ice and processed within 4 hours. Plasma was isolated through centrifugation at 1,200 × *g* for 10 min at 4°C, followed by a second centrifugation at 1,800 × *g* for 5 min to remove residual cellular debris. Plasma aliquots were snap-frozen on dry ice and stored at −80°C until RNA extraction. Following blood collection, craniotomy and tumor resection were performed under neuronavigation guidance. Resected tumor tissue was immediately snap-frozen in fresh medium until RNA extraction.

### RNA Extraction

RNA was extracted from frozen plasma using the miRNeasy Micro Kit (Qiagen) with minor modifications. Briefly, plasma was mixed with QIAzol Lysis Reagent, incubated 5 minutes, and extracted with chloroform (12,000 × *g*, 15 min, 4°C). The aqueous phase was combined with ethanol and glycogen, loaded onto RNeasy MinElute spin columns, washed sequentially with Buffer RWT, Buffer RPE, and 80% ethanol, and eluted in RNase-free water. Total RNA including small RNA was extracted from frozen bulk tumor tissue without additional dissection to enrich tumor cellularity using the miRNeasy Mini Kit (Qiagen).

### Small RNA Library Preparation and Sequencing

Small RNA libraries were prepared using the NEBNext Small RNA Library Prep Set for Illumina (New England Biolabs) with established protocol modifications.^23^ After adaptor ligation, reverse transcription was performed using ProtoScript II, followed by cDNA amplification (18-20 cycles). Libraries were purified with AMPure XP beads (Beckman Coulter), quantified using the Qubit HS dsDNA Assay (Invitrogen), and sequenced on an Illumina HiSeq 4000 platform (50-bp, single-end reads).

### Bioinformatics Analysis

Raw sequencing reads were trimmed to remove adaptor sequences and low-quality bases, then aligned to the human genome (GRCh38) using Bowtie.^24^^,^^25^ Genome alignment was used to assess mapping quality and exclude reads arising from non-miRNA small RNAs or repetitive elements. To accurately quantify mature miRNAs, whose short length and specific cleavage-derived sequences are not uniquely resolved by genome alignment, reads were independently aligned to miRBase, the curated reference database for canonical mature miRNA sequences.^26^^,^^27^ miRNA abundances were normalized as reads per million (RPM).^23^

To focus on tumor-derived signals, we first identified miRNAs with expression ≥10 RPM in tumor tissue.^28^^,^^29^ These tumor miRNAs were then examined in matched plasma samples collected before and after FUS. Based on their pre-FUS plasma levels, they were categorized as either below-threshold (plasma RPM <10) or above-threshold (plasma RPM ≥10).^30^ This classification provided a clear framework for determining whether sonobiopsy selectively enriches tumor-derived miRNAs that are typically undetectable in circulating blood.

From 199 miRNAs that were consistently expressed across all 11 tumor tissues (RPM ≥ 10), we then looked for miRNAs that were below threshold in pre-FUS plasma (RPM < 10), but rose above threshold (RPM ≥ 10) in plasma at 5, 10, or 30 min after FUS. We then selected miRNAs that met all of the following criteria:tumor RPM ≥ 10, pre-FUS plasma RPM < 10, and post-FUS plasma RPM ≥ 10 at any of the three time points in at least three patients. These selected miRNAs were used for further pathway analysis. DIANA-miRpath (v.4.0) was used to identify the associated signaling pathways associated with potential miRNA targets.^31^ Top 25 KEGG pathways were selected for dotplot based on merged *P* value and false discovery rate (FDR). The dotplot was generated using SRplot, an online platform for data analysis and visualization.^32^ This workflow enabled systematic characterization of the biological processes and signaling networks potentially regulated by miRNAs enriched through sonobiopsy.

### Statistical Analysis

**Figure 2.**
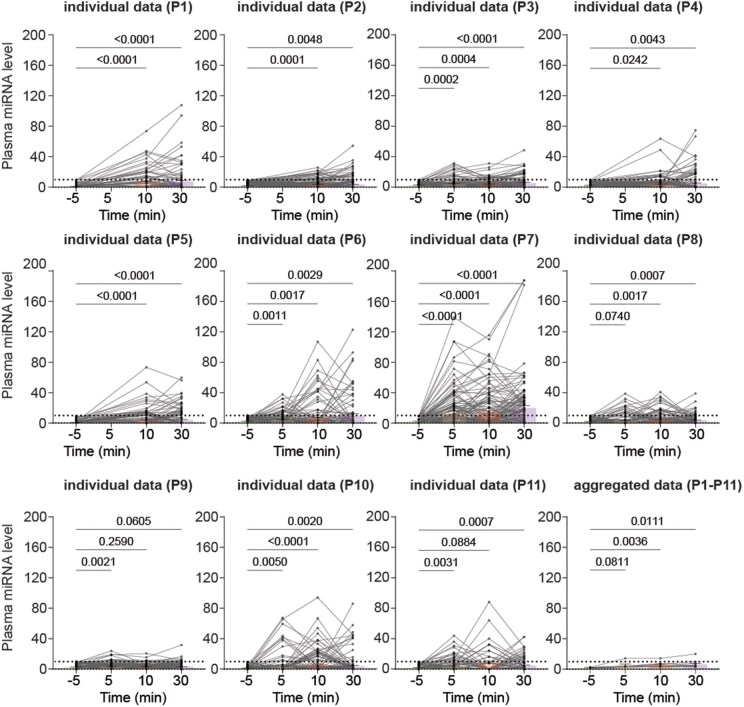
Plasma levels of below-threshold circulating miRNAs following sonobiopsy. The horizontal dashed line on each plot indicates the detection threshold (RPM = 10). For plots of individual patient: line plots show plasma levels of individual below-threshold miRNAs (pre-FUS RPM <10) at pre-FUS (5 min pre-FUS) and at 5, 10, and 30 min after FUS sonication. 5-minute post-FUS measurements were missing for four patients (P1, P2, P4, and P5) because their 5-min post-FUS plasma samples were fully used for other analyses beforehand and were not available for the miRNA analysis here. Statistical comparisons between pre-FUS and post-FUS time points were performed using repeated-measures one-way ANOVA with uncorrected Fisher’s least significant difference (LSD) test. For the aggregated plot of all patients: mean plasma levels of below-threshold miRNAs were quantified at pre-FUS and at 5, 10, and 30 min after FUS sonication across 11 patients. Each line represents the mean plasma levels of below-threshold miRNAs for an individual patient. Bar graphs show group means ± standard error of the mean (SEM) at each time point. Statistical comparisons between pre-FUS and each time point post-FUS were assessed using repeated-measures one-way ANOVA with uncorrected Fisher’s LSD test.

**Figure 3.**
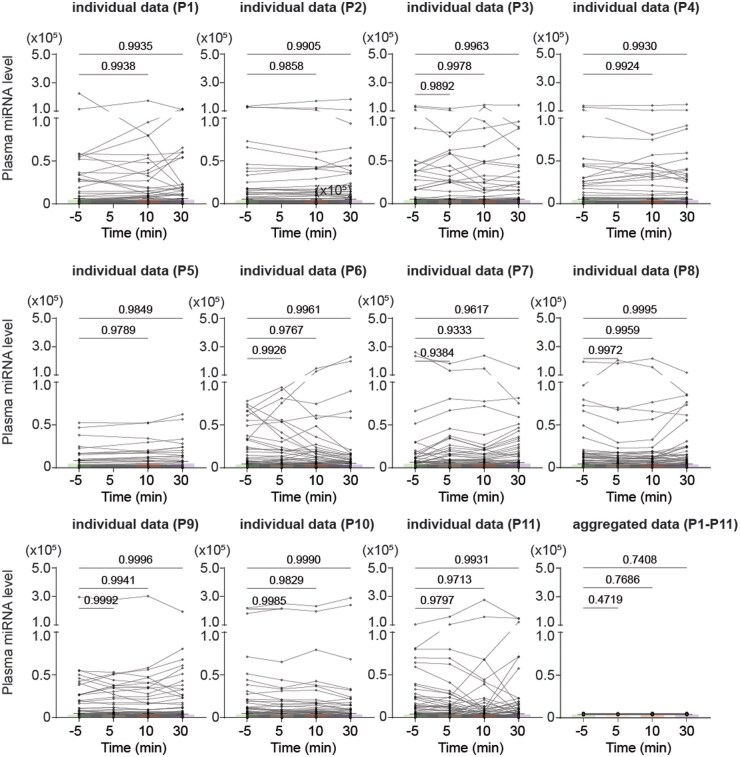
Plasma levels of above-threshold circulating miRNAs following sonobiopsy. For plots of individual patient: line plots show plasma levels of individual above-threshold miRNAs (pre-FUS RPM ≥10) at pre-FUS (5 minutes pre-FUS) and at 5, 10, and 30 min after FUS sonication. 5-minute post-FUS measurements were missing for four patients (P1, P2, P4, and P5) because their 5-min post-FUS plasma samples were fully used for other analyses beforehand and were not available for the miRNA analysis here. Statistical comparisons between pre-FUS and post-FUS time points were performed using repeated-measures one-way ANOVA with uncorrected Fisher’s least significant difference (LSD) test. For the aggregated plot of all patients: mean plasma levels of above-threshold miRNAs were quantified at pre-FUS and at 5, 10, and 30 min after FUS sonication across 11 patients. Each line represents the mean plasma levels of above-threshold miRNAs for an individual patient. Bar graphs show group means ± standard error of the mean (SEM) at each time point. Statistical comparisons between pre-FUS and each time point post-FUS were assessed using repeated-measures one-way ANOVA with uncorrected Fisher’s LSD test.

**Figure 4.**
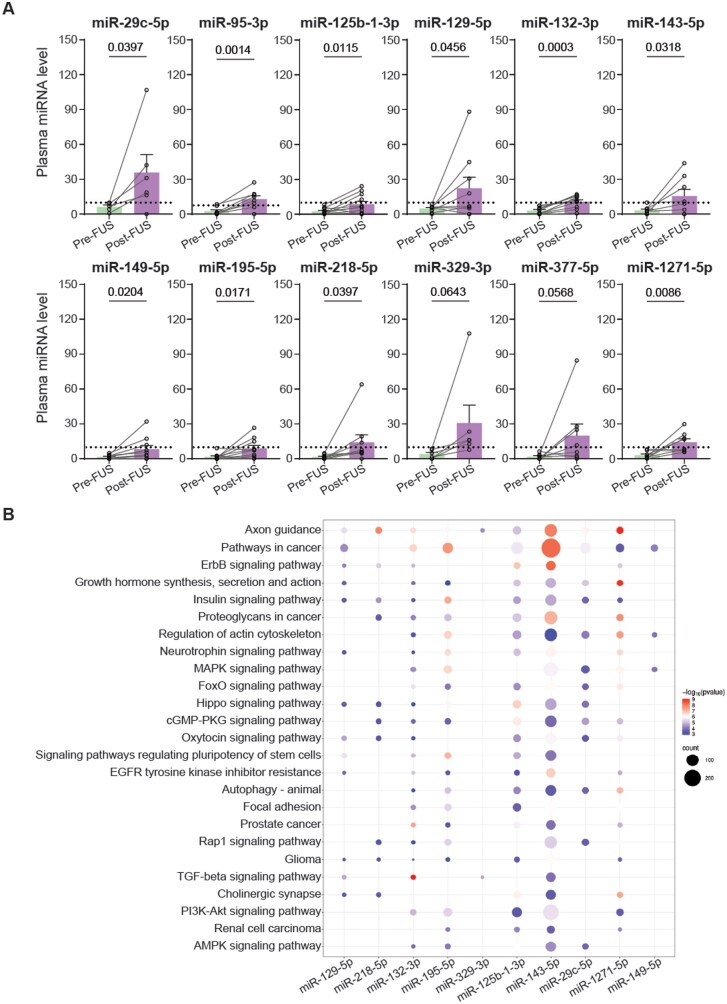
Below-threshold circulating miRNAs enriched with sonobiopsy are associated with cancer and glioma pathways. (A) Plasma levels of 12 below-threshold miRNAs that transitioned to above-threshold following sonobiopsy. Each line represents the plasma level of a specific miRNA for an individual patient. Pre-FUS levels were measured at 5 min before sonication, and post-FUS levels correspond to the highest value observed at 5, 10, or 30 min after FUS. Patients who already had above-threshold pre-FUS plasma levels (RPM ≥ 10) for a specific miRNA were excluded from the analysis for that miRNA. Bar graphs show group means ± standard error of the mean (SEM). The horizontal dashed line on each plot indicates the detection threshold (RPM= 10). Statistical comparisons between pre-FUS and each post-FUS time point were assessed using non-parametric t-tests. (B) KEGG pathway enrichment analysis of the 12 enriched miRNAs with DIANA-miRParh (v4.0). Pathway ranked by merged *P* value and false discovery value (FDR) and top 25 enriched KEGG pathways were ploted with SRplot.

We included 11 patients in this study. This sample size was considered sufficient for evaluating the feasibility of sonobiopsy in enriching circulating miRNAs. Because all patients underwent the same sonobiopsy procedure as part of the study protocol, randomization was not applicable, and blinding was not performed. Statistical analyses were conducted with GraphPad Prism (GraphPad, Boston, MA). Repeated measures one-way ANOVA with Fisher’s LSD test was used to compare post-FUS miRNA levels with pre-FUS for individual patients. *P* value for Figures 2 and 3 were two-tailed, and *P* value for Figure 4 were one-tailed, with *P < *.05 considered statistically significant.

## Results

### Sonobiopsy Enriches Below-Threshold Circulating miRNAs in Plasma

Across tumor samples from 11 glioma patients, we profiled all known human miRNAs using small RNA-seq. Applying a threshold of ≥10 RPM to define miRNA expression in tumor tissue, each tumor harbored an average of 390 expressed miRNAs, with 199 miRNAs consistently detected across all tumors. We next assessed whether these tumor-derived miRNAs could be detected in the matched plasma samples before and after FUS.

Below-threshold circulating miRNAs (pre-FUS plasma level RPM <10) showed robust, consistent enrichment following sonobiopsy (Figure 2). Pre-FUS plasma samples showed very low pre-FUS levels for these miRNAs across all patients (mean RPM: 0.71–2.37), reflecting their limited detectability under physiological conditions. After FUS, plasma levels of these miRNAs increased substantially at all time points, reaching 2.84–14.15 RPM at 5 min, 2.62–14.15 RPM at 10 min, and 2.86–19.89 RPM at 30 min, with the highest fold change among 11 patients being 6.2-fold at 5 min, 6.4-fold at 10 min, and 8.7-fold at 30 min. Repeated measures analysis confirmed significant increases relative to pre-FUS in all 11 patients (Figure 2, individual data plot).

Group-level averaged analysis further supported this pattern. When mean plasma levels of below-threshold miRNAs were averaged across all patients, sonobiopsy produced significant increases at 5-, 10-, and 30-min post-FUS compared with the pre-FUS (Figure 2, aggregated data plot). The average fold changes were 2.9 at 5 min, 3.1 at 10 min, and 3.6 at 30 min, reflecting a sustained, progressively increasing release of miRNAs throughout the sampling period. Together, these results demonstrate that sonobiopsy consistently and selectively increases the detectability of below-threshold miRNAs in the plasma of glioma patients, overcoming a significant limitation of conventional liquid biopsy for brain tumors.

### Sonobiopsy Does Not Increase Plasma Levels of Above-Threshold miRNAs

For above-threshold circulating miRNAs (pre-FUS plasma level RPM ≥10), pre-FUS plasma levels were substantially higher than those of below-threshold miRNAs, with mean RPM values ranging from 3,503 to 4,940 across patients. Unlike the pronounced increases observed for below-threshold miRNAs, plasma levels of above-threshold miRNAs remained stable after FUS sonication (Figure 3). Average post-FUS levels were 3,503–4,598 RPM at 5 min, 3,504–4,935 RPM at 10 min, and 3,503–4,935 RPM at 30 min, showing no meaningful deviation from pre-FUS.

Individual-patient analyses confirmed the absence of enrichment following sonobiopsy: above-threshold miRNAs exhibited flat temporal profiles with no significant differences between pre-FUS and any post-FUS time point (Figure 3, individual data plot). Group-level averaged analyses showed the same pattern. Mean plasma levels across the cohort remained unchanged at 5, 10, and 30 min after FUS (Figure 3, aggregated data plot).

Together, these findings demonstrate that sonobiopsy selectively enhances below-threshold miRNAs, while leaving above-threshold miRNAs unaffected. This selectivity indicates that FUS-BBBO does not induce a generalized increase in circulating miRNA levels, but instead preferentially mobilizes miRNAs that are constrained by the BBB and present near or below the detection threshold under pre-FUS conditions.

### Sonobiopsy-Enriched miRNAs Are Associated With Cancer- and Glioma-Related Pathways

To identify below-threshold miRNAs consistently enriched across the cohort, we focused on miRNAs that were robustly expressed in all 11 tumors (tumor RPM ≥10) yet exhibited below-threshold expression in pre-FUS plasma (RPM < 10). We then evaluated which of these tumor-derived miRNAs increased to above-threshold levels (RPM ≥ 10) in plasma after FUS. This cross-patient analysis revealed 12 miRNAs, including miR-29c-5p, miR-95-3p, miR-125b-1-3p, miR-129-5p, miR-132-3p, miR-143-5p, miR-149-5p, miR-195-5p, miR-218-5p, miR-329-3p, miR-377-5p, and miR-1271-5p, which transitioned from below to above detection threshold following FUS sonication (Figure 4A). The plasma levels of each of the 12 miRNAs for each patient at 5 min pre-FUS and at 5, 10, and 30 min post-FUS are listed in Table S2.

Pathway enrichment analysis of the 12 miRNAs was then conducted using DIANA-miRpath (v.4.0). Ten of these miRNAs (except miR95-3p and miR377-5p) and their top 25 KEGG pathways were associated with cancer and glioma pathways, as well as other pathways associated with glioma growth, invasion, and therapeutic resistance, including FoxO, MAPK, cGMP-PKG, PIK3-Akt, and EGFR tyrosine kinase inhibitor resistance (Figure 4B).

Collectively, these findings indicate that sonobiopsy selectively mobilized tumor-derived miRNAs that participate in molecular networks known to associated with glioma pathogenesis, supporting their biological and diagnostic relevance.

## Discussion

This study provides the first clinical evidence that circulating miRNAs can serve as noninvasive biomarkers enriched by sonobiopsy. By demonstrating that FUS combined with microbubbles significantly increases plasma levels of below-threshold miRNAs, our work establishes the feasibility of using sonobiopsy to expand the molecular information obtainable from blood in patients with brain tumors. These findings represent an important step toward overcoming one of the central challenges in liquid biopsy for neuro-oncology: the extremely low abundance of brain tumor-derived biomarkers in blood circulation.

Liquid biopsy has transformed the management of cancers outside the brain, but its utility in glioma has been limited by the restrictive BBB and the low rate of biomarker shedding from intracranial tumors.^6^^,^^7^ Prior efforts in blood-based glioma diagnostics have focused largely on improving analytical sensitivity^33^; however, assay sensitivity alone cannot overcome the intrinsic scarcity of tumor-derived biomarkers entering the bloodstream. Sonobiopsy provides a fundamentally different solution. Using FUS to actively increase the release of tumor-derived molecules into circulation, sonobiopsy complements existing detection platforms and enables access to molecular signatures that would otherwise remain below the detection threshold.

The enrichment of tumor-derived miRNAs is particularly meaningful given their well-established roles in glioma biology. In this study, sonobiopsy increased plasma levels of below-threshold miRNAs by up to 8.7-fold, whereas above-threshold miRNAs remained unchanged. This differential response likely reflects biological and biophysical constraints governing biomarker release. Below-threshold miRNAs, which circulate at near-undetectable levels under pre-FUS conditions due to limited BBB permeability, benefit disproportionately from FUS-enhanced transport of tumor-derived biomarkers into the bloodstream; even modest increases in tumor-derived release led to measurable rises in plasma levels. By contrast, above-threshold miRNAs may be predominantly derived from systemic sources (e.g., blood immune cells and endothelial compartments) and maintain steady-state concentrations that mask small contributions from the tumor, leaving their post-FUS levels unchanged.

Importantly, the miRNAs enriched following sonobiopsy, including miR-29c-5p, miR-125b-1-3p, miR-129-5p, miR-132-3p, miR-143-5p, miR-149-5p, miR-195-5p, miR-218-5p, miR-329-3p, and miR-1271-5p, were associated with pathways invovled in glioma growth, invasion, and therapeutic resistance. Among them, miR-29c-5p, miR-125b-1-3p, miR-129-5p, miR-143-5p, miR-149-5p, miR-195-5p, miR-218-5p, and miR-329-3p act as tumor suppressors, inhibiting cell proliferation, invasion, and angiogenesis in glioma cells.^34-42^ miR-29c-5p, miR-129-5p, and miR-218-5p are often down-regulated in glioma and high expression of these miRNAs is often associated with better overall survival in patients with glioma.^36^^,^^43^^,^^44^ Restoration of miRNAs like miR-29c-5p and miR-195-5p could overcome resistance to common treatments like Temozolomide.^45^^,^^46^ In contrast, miR-132 promotes gliomagenesis by enhancing caveolae-mediated transcellular transport in glioma endothelial cells.^47^ Future studies are needed to validate whether sonobiopsy-enriched miRNAs could serve as noninvasive diagnosis markers for glioma.

Our results also build upon the growing body of evidence establishing sonobiopsy as a platform technology capable of enriching multiple classes of brain tumor biomarkers. In our prior work, we demonstrated enhanced detection of tumor-derived DNA biomarkers, including mono-nucleosomal cfDNA, patient-specific ctDNA, and TERT promoter mutations.^12^ In a retrospective FUS-BBBO clinical trial, Meng *et al.* have reported increases in cfDNA, neuron-derived extracellular vesicles, brain-specific proteins, and DNA methylation signatures following FUS-BBBO. Recent reported drug-delivery trials using FUS in glioma patients found that disease state correlated closely with changes in plasma cfDNA over time and plasma extracellular vesicle protein release predicted outcomes following paclitaxel treatment, suggesting the potential for noninvasive treatment monitoring.^9^^,^^10^ Here, we extend these findings to miRNAs, collectively demonstrating that sonobiopsy can increase the availability of diverse biomarker modalities, addressing a long-standing barrier in brain tumor liquid biopsy.

Our analysis focused on miRNAs that were detectable in tumor tissue but below threshold in pre-FUS plasma. The consistent, temporally aligned increases of these previously below-threshold miRNAs in pre-FUS plasma after spatially targeted FUS support an association with the tumor tissue. Here, we analyzed miRNAs from bulk tumor tissue without additional dissection to enrich for tumor cells, meaning that both tumor and non-tumor components were included in the small RNA sequencing. As a result, we cannot precisely determine the relative contribution of non-tumor cells to the overall miRNA signal in the tumor samples. To more definitively establish the tumor origin of miRNAs that were below the detection threshold in pre-FUS plasma but elevated after FUS, future sonobiopsy studies need to incorporate tumor-specific miRNA profiling approaches. These may include microdissection to selectively increase tumor cellularity, miRNA profiling of healthy brain tissue as a control, and single-cell mRNA and miRNA profiling to resolve cell-type-specific miRNA expression.

We noticed that some of the below-threshold miRNAs increased in plasma following FUS, whereas others did not. These findings suggest that only specific subsets of miRNAs are enriched in plasma by FUS-BBBO. Several potential explanations related to the spatial and temporal properties of FUS may account for this discrepancy. First, different miRNAs likely have distinct release dynamics following FUS-BBBO and different clearance kinetics, such that a given sampling time may capture transient peaks for some miRNAs but miss others. Second, miRNAs may be heterogeneously distributed within the tumor, such that a single FUS-BBBO event releases only a subset of FUS-targeted tumor tissue miRNAs into the circulation. Therefore, not all cancer-related miRNAs would necessarily be elevated in plasma after FUS. Lastly, FUS targeted at tumor tissue may also release a set of miRNAs from non-tumor cells into the blood; therefore, not all miRNAs that increase in plasma after FUS are necessarily cancer-associated, which also relates to the above limitation. Future sonobiopsy studies need to implement prolonged blood collection windows and target larger tumor volumes to potentially ameliorate this discrepancy.

The sample size was modest and derived from a single center, consistent with the exploratory nature of a first-in-human trial. Larger, multicenter studies will be needed to validate these findings, define optimal sampling windows, and assess clinical utility. In addition, while we identified a miRNA signature enriched by sonobiopsy, functional studies and correlations with treatment response or clinical outcomes were beyond the scope of this work. Future research should incorporate multimodal liquid biopsy analyses and evaluate the value of sonobiopsy for longitudinal monitoring, early recurrence detection, and treatment stratification.

In conclusion, this study highlights a key innovation: circulating miRNAs, which have long been viewed as promising but difficult-to-detect biomarkers for brain tumors, can be sensitively and noninvasively enriched using sonobiopsy. By enabling spatially targeted, temporally controlled release of tumor-derived molecular information into the bloodstream, sonobiopsy overcomes a fundamental barrier in brain tumor liquid biopsy. The feasibility and biological insights demonstrated here provide a strong foundation for advancing sonobiopsy toward a clinically impactful diagnostic tool for gliomas and, potentially, other neurological diseases.

## Supplementary Material

vdag136_Supplementary_Data

## Data Availability

The miRNA sequencing data of patient tumors and plasmas in this study have been deposited in the NCBI SRA (PRJNA1009135).
