# Supplementary material for: Sonobiopsy for enrichment of circulating microRNAs in glioma patients
Source: Neurooncol Adv. 2026 May 22;8(1):vdag136. doi: 10.1093/noajnl/vdag136 (PMC13271035; doi:10.1093/noajnl/vdag136)
Supplement: vdag136_Supplementary_Data [file vdag136_supplementary_data.zip › Supplementary Table 1.docx]

**Supplementary Table 1: Inclusion and exclusion criteria**

| **Inclusion Criteria:** |
| --- |
| - Must be newly diagnosed with a lesion in the brain with imaging characteristics consistent with high-grade glioma. Scan must have occurred no more than 28 days prior to enrollment. - Lesion must be > 3 cm in maximal dimension on MRI. - Lesion must be in the supratentorial space within 5 cm of the cortical surface. - Lesion must be gadolinium-enhancing. - Low-grade tumors and metastatic tumors - Recurrent brain tumors and/or radiation necrosis - Must be planning to undergo surgical resection of the tumor. - Must be at least 18 years old. |
| **Exclusion Criteria:** |
| - Contraindication to MRI. - Previous cranial surgery. - Previous history of cancer and/or cancer treatments. - Coagulopathy within 14 days of enrollment defined as PT/PTT outside of normal parameters and platelets < 100,000/mcL. - Physical skull defect of any kind. - Ferrous material in the scalp or skull. - Scalp or skin disease that limits contact with the ultrasound probe. - Enrolled in another clinical trial where intervention is administered prior to surgery. - Known hypersensitivity to polyethylene glycol. - Known unstable cardiopulmonary condition (e.g., acute myocardial infarction, acute coronary artery syndromes, worsening or unstable congestive heart failure, serious ventricular arrhythmias). |
